# Three new species of *Argyra* from China (Diptera, Dolichopodidae, Diaphorinae)

**DOI:** 10.3897/zookeys.772.25406

**Published:** 2018-07-06

**Authors:** Qilemoge, Mengqing Wang, Ding Yang

**Affiliations:** 1 Department of Entomology, College of Plant Protection, China Agricultural University, Beijing 100193, China; 2 Institute of Plant Protection, Chinese Academy of Agricultural Sciences, Beijing 100193, China

**Keywords:** Diptera, Dolichopodidae, Diaphorinae, *Argyra*, China, new species

## Abstract

Previously, there were ten known species in the genus *Argyra* Macquart from China. In this paper, the following three species from Sichuan Province of China are described as new to science: *Argyra longicornis* **sp. n.**, *Argyra pingwuensis* **sp. n.** and *Argyra sichuanensis* **sp. n.** A key to the known species of *Argyra* from China is provided.

## Introduction

*Argyra* Macquart belongs to the subfamily Diaphorinae (Dolichopodidae) with 117 known species and three fossil species from around the world (Yang et al. 2006, Yang et al. 2011, Grichanov 2017). The following ten species were known to occur in China: *Argyra arrogans* Takagi, 1960, *A. nigripilosa* Yang & Saigusa, 2002, *A. pallipilosa* Yang & Saigusa, 2002, *A. pseudosuperba* Hollis, 1964, *A. sinensis* Yang & Grootaert, 1999, and *A. vanoyei* (Parent, 1926) from the Oriental realm and *A. beijingensis* Wang & Yang, 2004, *A. serrata* Yang & Saigusa, 2002, *A. tibetensis* Wang, Chen & Yang, 2015, and *A. xiaolongmensis* Wang & Yang, 2011 from the Palaearctic realm (Yang et al. 2011).

Sichuan province is located in southwest China, covering an area of 48,000 square kilometers. The topography of Sichuan varies from mountains to basins, undulating with plateaus and hills. Usually, this region has typical subtropical monsoon climate. These patterns together contribute to the rich species diversity of insects. Here *Argyra* is reported for the first time from Sichuan with three new species.

## Materials and methods

The specimens upon which this study is based, were collected from Sichuan province of China in 2016 by sweep nets and yellow pan traps. After the genitalia was dissected, it was placed in a small centrifuge tube containing glycerin and stored in glass tube containing the corresponding specimen (all specimens were stored in alcohol) and deposited in the Entomological Museum of China Agricultural University (**CAU**), Beijing. Morphological terminology for adult structures mainly follows McAlpine (1981). Terms for the structures of the male genitalia follow Cumming and Wood (2009). The following abbreviations are used:

**acr** acrostichal bristle (s),

**ad** anterodorsal bristle (s),

**av** anteroventral bristle (s),

**cer** cercus,

**CuAx ratio** length of m-cu / length of distal portion of CuA,

**dc** dorsocentral bristle (s),

**hyp** hypandrium,

**LI** fore leg,

**LII** mid leg,

**LIII** hind leg,

**npl** notopleural bristle (s),

**oc** ocellar bristle (s),

**pa** postalar bristle (s),

**pd** posterodorsal bristle (s),

**pvt** postvertical bristle (s),

**sa** supraalar bristle (s),

**sc** scutellars,

**sur** surstylus,

**vt** vertical bristle (s).

## Taxonomy

### Argyra

Taxon classificationAnimaliaDipteraDolichopodidae

Macquart, 1834

Argyra Macquart, 1834: 456. Type species: Musca diaphana Fabricius, 1775.

#### Diagnosis.

Vertex somewhat excavated, occiput slightly concave medially. Ocellar tubercle distinct with two long oc and two short posterior hairs. Antennal scape bare in subgenus Leucostola and with dorsal hairs in subgenus Argyra; first flagellomere short to elongate; arista dorsal to subapical. Propleuron with 1 pale bristle on upper portion and 1–2 pale or black bristles on lower portion. Hind coxa with 2–6 strong or weak vertical outer bristles. Mid and hind femora without preapical bristle. CuAx ratio 0.4–0.55. Surstylus long or short, with dorsal and ventral lobes. Hypandrium long, bent medially, apically rounded.

##### Key to species (males) of *Argyra* from China

**Table:** 

1	Antennal scape bare [subgenus Leucostola Loew]	**2**
–	Antennal scape with dorsal hairs [subgenus Argyra Macquart]	**3**
2	Fore coxa with black hairs and bristles; mid tibia with 2 av; hypopygium with strong bristles; dorsal lobe of surstylus narrow from middle to tip (Yang et al. 2011, fig. 718b)	**A. (L.) sinensis Yang & Grootaert**
–	Fore coxa with yellow hairs and bristles; mid tibia without av; hypopygium without strong bristles; dorsal lobe of surstylus narrow apically (Yang et al. 2011, fig. 719a)	**A. (L.) vanoyei Parent**
3	Palpus yellow	**4**
–	Palpus black	**6**
4	Male fore tarsus modified with tarsomeres 2–4 shortened and thickeneded	**5**
–	Male fore tarsus normal, tarsomeres 2–4 neither shortened nor thickeneded	**A. (A.) serrata Yang & Saigusa**
5	All coxae with yellow hairs and bristles; hind femur with dark brown tip; first flagellomere 2.5–3.0 times longer than wide (Yang et al. 2011, fig. 716a)	**A. (A.) pallipilosa Yang & Saigusa**
–	All coxae with black hairs and bristles; hind femur wholly yellow; first flagellomere 2.1 times longer than wide (Yang et al. 2011, fig. 715a)	**A. (A.) nigripilosa Yang & Saigusa**
6	M with strong Z-bend (Yang et al. 2011, fig. 716d)	**A. (A.) pseudosuperba Hollis**
–	M with weak bend	**7**
7	Acr absent	**A. (A.) tibetensis Wang, Chen & Yang**
–	Acr present	**8**
8	Surstylus incised, separated into paired lobes to about midlength (Fig. 8)	**9**
–	Surstylus deeply incised, separated into paired lobes to base (Figs 11, 13)	**10**
9	First flagellomere much elongated, 3.8 times longer than wide (Figs 2, 7)	**A. (A.) longicornis sp.n.**
–	First flagellomere elongated, 2.2 times longer than wide (Yang et al. 2011, fig. 713a)	**A. (A.) arrogans Takagi**
10	3 acr irregularly arranged anteriorly, 3 acr in single row posteriorly; hind femur and tibia wholly yellow	**A. (A.) xiaolongmensis Wang & Yang**
–	6–7 acr irregularly arranged; hind femur and tibia not as above	**11**
11	All femora black, only apical 1/4 of fore femur yellow; apical 1/3 of hind tibia black (Fig. 5); fore tarsomere 2 slightly thickened with short thick bristles; dorsal lobe of surstylus strip-like, apical unobviously inflated (Fig. 13)	**A. (A.) sichuanensis sp. n.**
–	Femora and tibiae not as above; fore tarsomere 2 not thickened, without short thick bristles; dorsal lobe of surstylus basally narrow, apical obviously inflated	**12**
12	Abdominal tergites 1–2 with large yellow lateral spot; basal 1/3 of fore and mid femora and apical 1/3 of hind femur black; apical 1/4 of hind tibia black (Fig. 3); ventral lobe of surstylus ventrally with 1 protuberance (Fig. 11)	**A. (A.) pingwuensis sp. n.**
–	Only abdominal tergite 2 with large yellow lateral spot; only hind femur with apical 1/5 black; basal 1/5 and apical 1/3 of hind tibia black; ventral lobe of surstylus without protuberance (Yang et al. 2011, fig. 714; Wang et al. 2004, fig. 2)	**A. (A.) beijingensis Wang & Yang**

### Argyra longicornis sp. n.

Taxon classificationAnimaliaDipteraDolichopodidae

http://zoobank.org/965F9189-4336-4A44-83C2-131D3BDA424E

Figs 1–2 , 7–9

#### Diagnosis.

First flagellomere much elongated, 3.8 times longer than wide; arista with basal segment 0.2 times as long as apical segment. Fore coxa yellow, mid and hind coxae black; hind femur black apically; fore tarsus yellow, mid tarsus from tip of tarsomere IV onward black. Hind tibia with 12 av; hind tarsomere I with six dorsal bristles and eight ventral bristles. CuAx ratio 0.52. Abdominal tergites 2–3 with large yellow lateral spot. Surstylus shallowly incised apically; dorsal lobe thumb-like; ventral lobe finger-like; cercus foliate.

#### Description.

**Figures 1–6.**
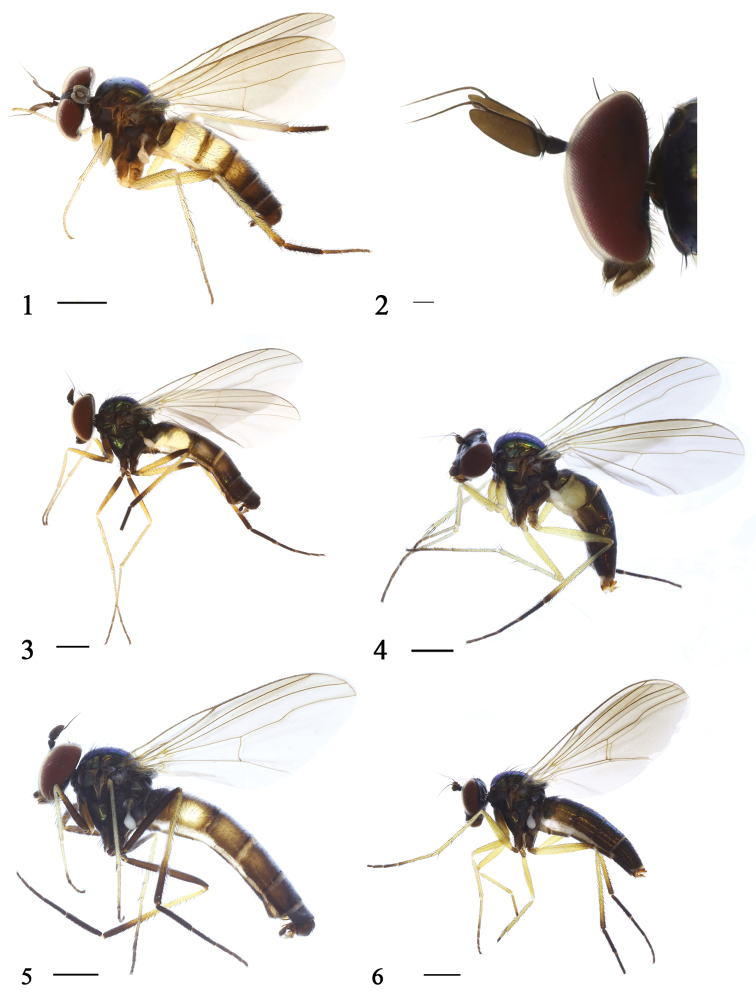
Habitus, lateral view. **1** *Argyra longicornis* sp. n. Male **2** *Argyra longicornis* sp. n. Male Antenna **3** *Argyra pingwuensis* sp. n. Male **4** *Argyra pingwuensis* sp. n. Female **5** *Argyra sichuanensis* sp. n. Male **6** *Argyra sichuanensis* sp. n. Female. Scale bars: 1 mm.

Male (Fig. 1). Body length 5.2 mm. Wing length 5.4 mm.

**Figures 7–9.**
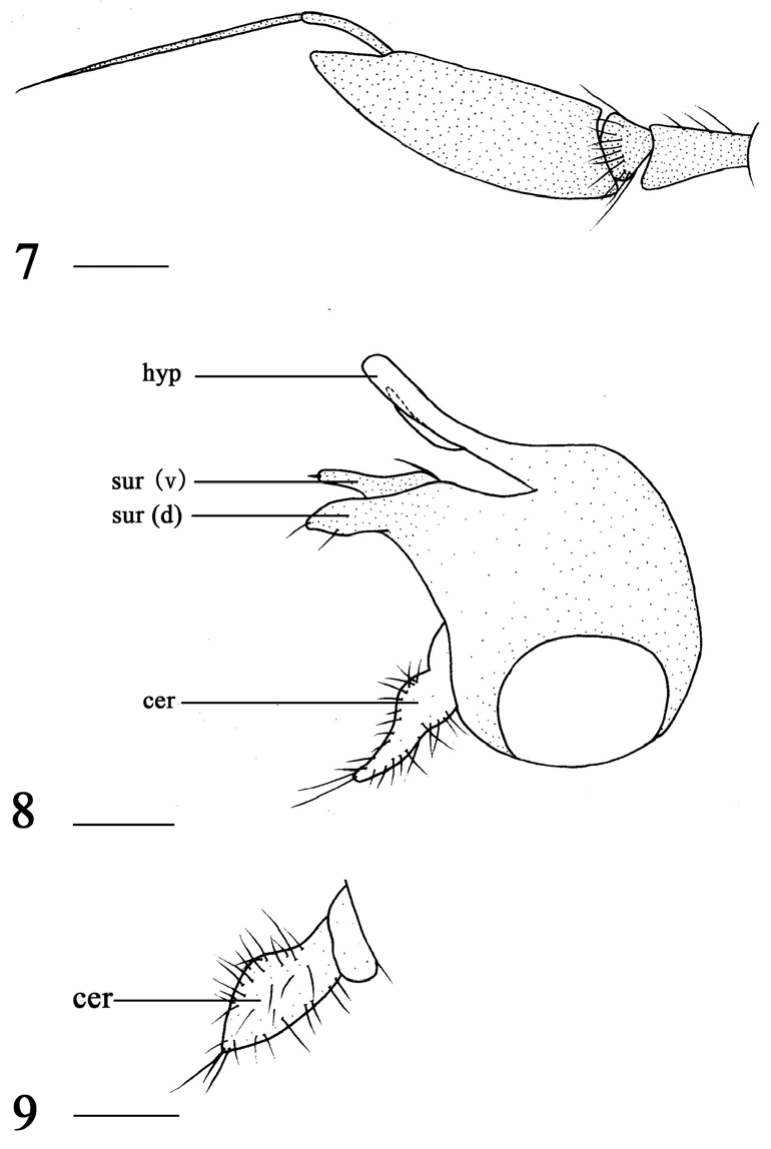
*Argyra longicornis* sp. n. male. **7** antenna, lateral view **8** genitalia, lateral view **9** cercus, lateral view. Abbreviations: hyp = hypandrium, sur (v) = ventral lobe of surstylus, sur (d) = dorsal lobe of surstylus, cer = cercus. Scale bars: 0.2 mm.

Head metallic green with pale gray pruinescence. Hairs and bristles on head black. Face brown with pale gray pruinescence, width of face equal to length of first flagellomere. Upper postocular bristles black, middle and lower postocular bristles yellow. Two oc, two vt, two pvt. Antenna (Figs 2, 7) brown; scape with black hairs, pedicel with circlet of black hairs; first flagellomere much elongated, 3.8 times longer than wide, apically slightly sharp; arista brown, indistinctly pubescent, basal segment 0.2 times as long as apical segment. Proboscis brown with black hairs; palpus black with black hairs.

Thorax metallic green with pale gray pruinescence. Hairs and bristles on thorax black. Five strong dc, four acr, two strong npl, one strong sa, two strong pa; scutellum with two pairs of sc. Propleuron with one pale bristle. Fore coxa yellow, mid and hind coxae black; fore and mid femora yellow, hind femur black apically; fore and mid tibiae yellow, hind tibia black apically; fore tarsus yellow, mid tarsus from tip of tarsomere IV onward black, hind tarsus entirely black. Hairs and bristles on legs black. Fore coxa with three bristles, mid coxa with three bristles and hind coxa with three black outer bristles. All femora with two rows of long black ventral bristles, length of bristles equal to width of femur. Fore tibia with five ad, six pd, three av, and three apical bristles; mid tibia with two ad, four pd, three av, and five apical bristles; hind tibia with four ad, five pd, 12 av, and two apical bristles. Hind tarsomere 1 with six dorsal bristles and eight ventral bristles. Relative lengths of tibia and 5 tarsomeres LI 3.5 : 2.4 : 0.7 : 0.5 : 0.3 : 0.4; LII 4.8 : 2.1 : 1.0 : 0.9 : 0.6 : 0.4; LIII 6.7 : 2.3 : 0.9 : 0.9 : 0.5 : 0.3. Wing hyaline, veins black; costal callus indistinct; M bent medially, M and R_4+5_ parallel apically; CuAx ratio 0.52. Squama yellow with black hairs. Halter yellow.

Abdomen metallic green with pale gray pruinescence, except tergites 2–3 with yellow lateral spot. Hairs and bristles on abdomen black. Male genitalia (Figs 8–9): Surstylus on epandrium shallowly incised apically, extending to midlength; dorsal lobe brown, thumb-like, apically sharp, with two bristles; ventral lobe brown, finger-like, basally wide with one long strong bristle, apically sharp with one bristle. Cercus foliated, long, basally wide, apically slightly acute with distinct bristles. Hypandrium yellow, long and slightly bent medially.

Female. Unknown.

#### Types.

Holotype male, CHINA, Sichuan, Chongzhou, Jiguanshanxian, Anzihe Nature Reserve; 1690 m; collected by sweeping nets in grassland; 2016.VII.30, Yuqiang Xi.

#### Distribution.

Oriental realm: China (Sichuan).

#### Remarks.

The new species is somewhat similar to *A. arrogans* Takagi, but can be separated from the latter by the following features: first flagellomere much elongated, 3.8 times longer than wide (Figs 2, 7); fore coxa yellow, mid and hind coxae black (Fig. 1); ventral lobe of surstylus with one long strong bristle at base; cercus foliated (Fig. 9). In *A. arrogans*, the first flagellomere is as elongate, 2.1 times longer than wide; all coxae are black; the ventral lobe of the surstylus has two long strong bristles at base; and the cercus is nearly quadrate with the sharp apex (Yang et al. 2011: 1106, fig. 713a–b).

#### Etymology.

The specific name refers to the elongated first flagellomere.

### Argyra pingwuensis sp. n.

Taxon classificationAnimaliaDipteraDolichopodidae

http://zoobank.org/B6D62008-F653-4490-A6D5-7654CD5B25DA

Figs 3–4 , 10–11

#### Diagnosis.

First flagellomere 2.1 times longer than wide; arista with basal segment 0.18 times as long as apical segment. All coxae black; basal 1/3 of fore and mid femora black, apical 1/3 of hind femur black; apical 1/4 of hind tibia black, av absent. CuAx ratio 0.43. Abdominal tergites 1–2 with yellow lateral spot. Surstylus deeply incised apically; dorsal lobe basally thin, apically inflated obviously; ventral lobe finger-like, ventrally with one protuberance.

#### Description.

Male (Fig. 3). Body length 6.2–6.5 mm. Wing length 5.3–5.5 mm.

**Figures 10–11.**
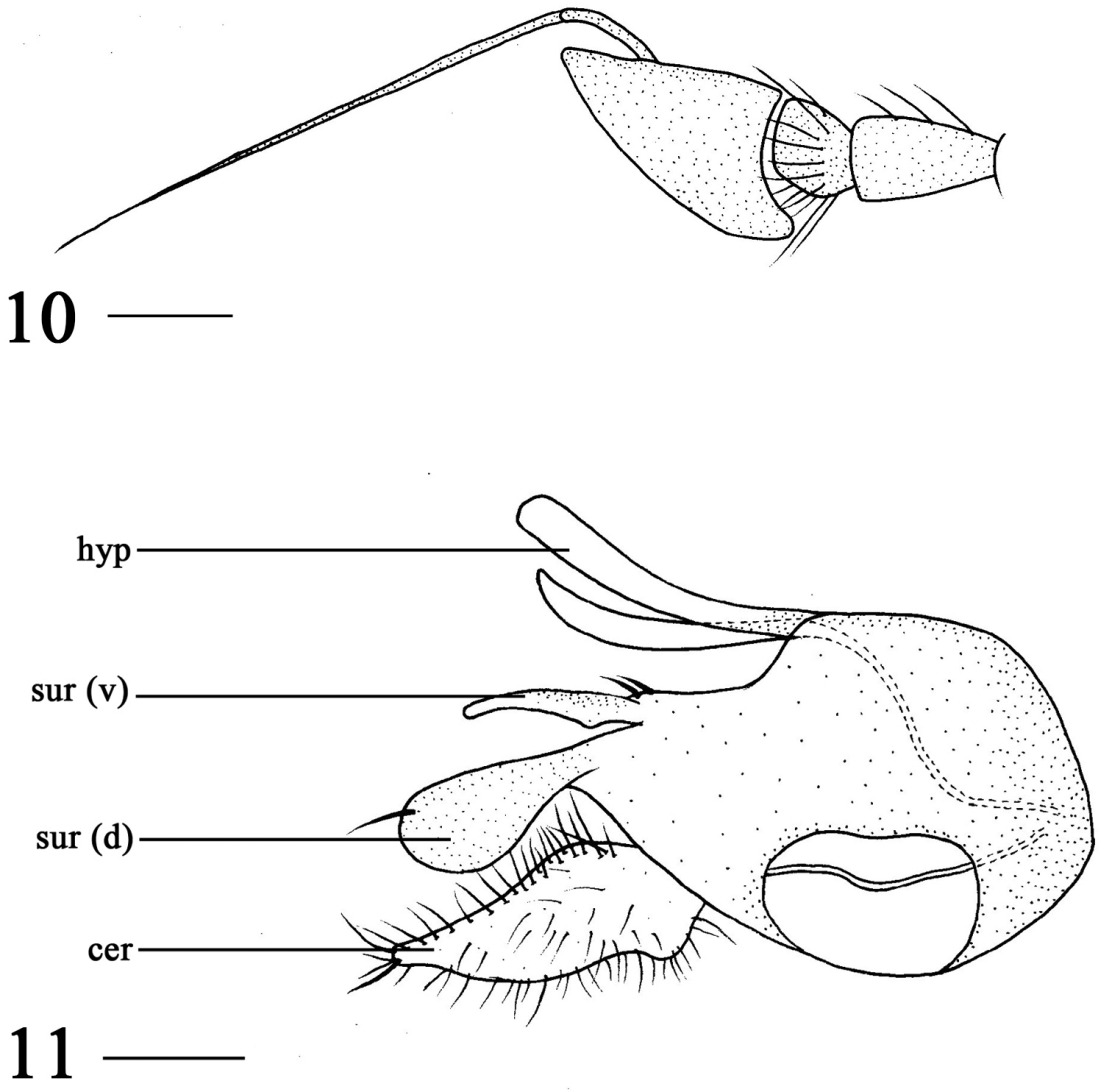
*Argyra pingwuensis* sp. n. male. **10** antenna, lateral view **11** genitalia, lateral view. Abbreviations: hyp = hypandrium, sur (v) = ventral lobe of surstylus, sur (d) = dorsal lobe of surstylus, cer = cercus. Scale bars: 0.2 mm.

Head metallic green with pale gray pruinescence. Hairs and bristles on head black. Face brown with pale gray pruinescence, width of face equal to length of first flagellomere. Upper postocular bristles black, middle and lower postocular bristles yellow. Two oc, two vt, two pvt. Antenna (Fig. 10) brown; scape with black hairs, pedicel with circlet of black hairs; first flagellomere elongated, 2.1 times longer than wide, apically slightly sharp; arista brown, indistinctly pubescent, basal segment 0.18 times as long as apical segment. Proboscis brown with brown hairs; palpus black with black hairs.

Thorax metallic green with pale gray pruinescence. Hairs and bristles on thorax black. Five strong dc, six acr, two strong npl, one strong sa, two strong pa; scutellum with two pairs of sc. Propleuron with one pale bristle. Legs with all coxae black; basal 1/3 of fore and mid femora black, apical 1/3 of hind femur black; fore and mid tibiae yellow, apical 1/4 of hind tibia black; fore and mid tarsi from tip of tarsomere 1 onward black, hind tarsus entirely black. Hairs and bristles on legs black. Fore coxa with two bristles, mid coxa with five bristles, hind coxa with two black outer bristles. All femora with two rows of long black ventral bristles, twice as long as width of femur. Fore tibia with one ad, two pd, and three apical bristles; mid tibia with two ad, four pd, three av, and five apical bristles; hind tibia with eight ad, eight pd, av absent and three apical bristles. Relative lengths of tibia and 5 tarsomeres LI 3.2 : 2.3 : 0.5 : 0.4 : 0.2 : 0.3; LII 5.2 : 3.1 : 1.2 : 1.0 : 0.6 : 0.4; LIII 6.7 : 2.4 : 1.7 : 1.2 : 0.7 : 0.5. Wing hyaline, veins black; costal callus indistinct; M bent medially, M and R_4+5_ parallel apically; CuAx ratio 0.43. Squama yellow with brown hairs. Halter yellow.

Abdomen metallic green with pale gray pruinescence, except tergites 1–2 with yellow lateral spot. Hairs and bristles on abdomen black. Male genitalia (Fig. 11): Surstylus deeply incised apically; dorsal lobe brown, basally thin, apically inflated obviously, with one long bristle; ventral lobe yellow, except dorsal surface brown, finger-like, basally wide, with two bristles, ventrally with protuberance. Cercus long, basally wide, apically slightly acute with distinct bristles. Hypandrium yellow, long and slightly bent medially.

Female (Fig. 4). Body length 5.4–5.6 mm. Wing length 5.4–5.6 mm. Similar to male except: Fore coxa yellow except basally black and all femora yellow. Abdomen tergite 2 with yellow lateral spot.

#### Types.

Holotype male, CHINA, Sichuan, Pingwu, Wanglang National Nature Reserve; 2930m; collected by sweeping nets in grassland; 2016.VIII.3, Yuqiang Xi. Paratypes: three males, four females, same data as holotype; four males, one female, CHINA, Sichuan, Pingwu, Wanglang National Nature Reserve; collected by yellow pan traps in grassland; 2016.VIII.5, Yuqiang Xi.

#### Distribution.

Oriental realm: China (Sichuan).

#### Remarks.

The new species is somewhat similar to *A. beijingensis* Wang & *Y*ang, but can be separated from the latter by the following features: fore and mid femora black at base 1/3, hind femur black at apical 1/3, hind tibia black at apical 1/4; dorsal lobe of surstylus with one long bristle. In *A. beijingensis*, the ventral surface of the fore and mid femora are black at base, the hind femur is black at apical 1/5; the dorsal lobe of the surstylus has three bristles (Wang et al. 2004: 386, fig. 2; Yang et al. 2011: 1106, fig. 714).

#### Etymology.

The specific name refers to the type locality, Pingwu.

### Argyra sichuanensis sp. n.

Taxon classificationAnimaliaDipteraDolichopodidae

http://zoobank.org/42AB7B4F-987F-44B5-B942-577D6B11E1AC

Figs 5–6 , 12–13

#### Diagnosis.

First flagellomere 1.92 times longer than wide; basal segment of arista 0.18 times as long as apical segment. All coxae black; all femora black, except apical 1/4 of fore femur yellow; hind tibia without av; fore tarsomere II slightly thickened with short thick bristles. CuAx ratio 0.48. Abdominal tergite 2 with large yellow lateral spot. Surstylus deeply incised apically; dorsal lobe nearly strip-like, ventral lobe finger-like, basally wide.

#### Description.

Male (Fig. 5). Body length 6.9–7.1 mm. Wing length 5.4–5.6 mm.

**Figures 12–13.**
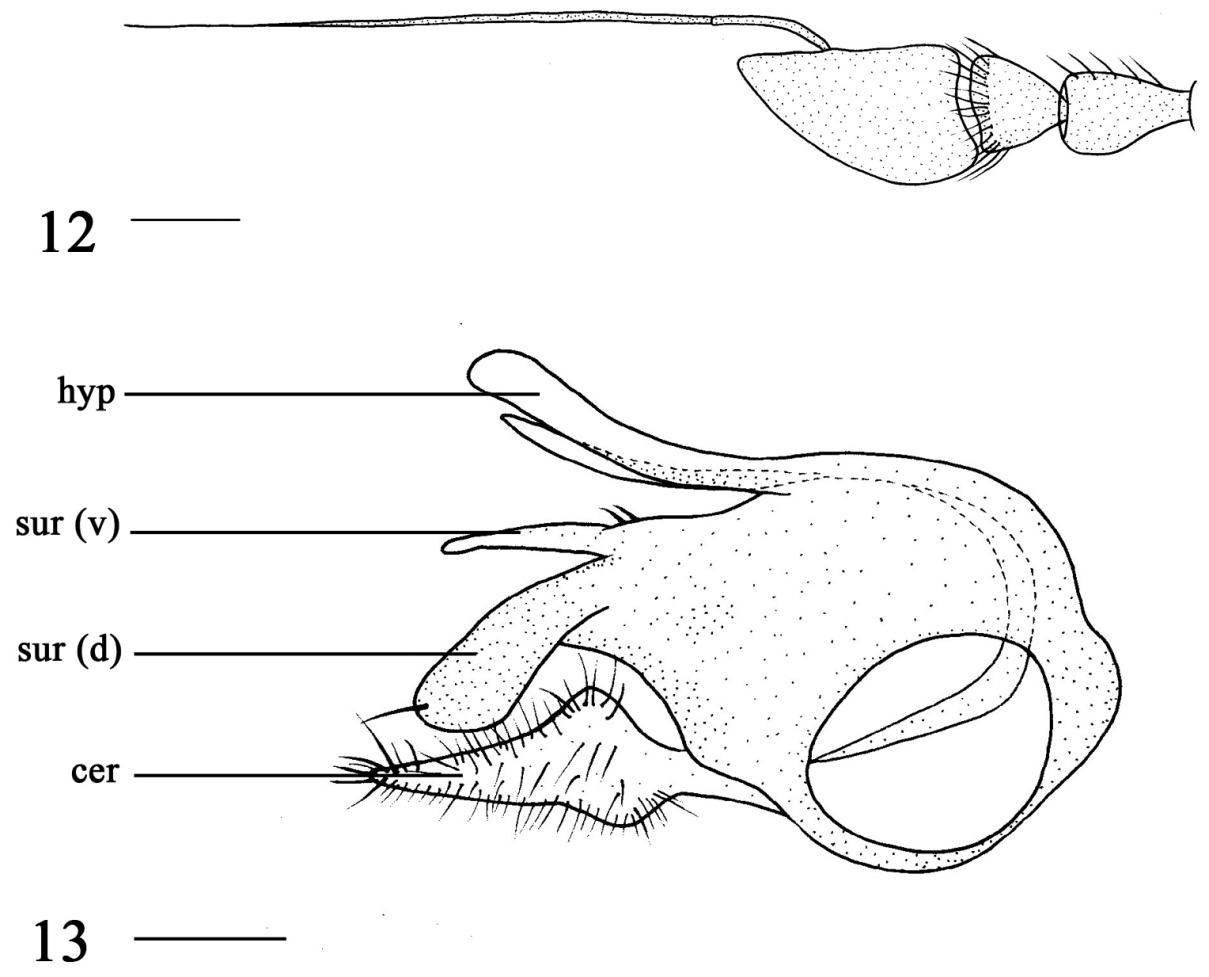
*Argyra sichuanensis* sp. n. male. **12** antenna, lateral view **13** genitalia, lateral view. Abbreviations: hyp = hypandrium, sur (v) = ventral lobe of surstylus, sur (d) = dorsal lobe of surstylus, cer = cercus. Scale bars: 0.2 mm.

Head metallic green with pale gray pruinescence. Hairs and bristles on head black. Face brown with pale gray pruinescence, width of face equal to length of first flagellomere. Upper postocular bristles black, middle and lower postocular bristles yellow. Two oc, two vt, two pvt. Antenna (Fig. 12) black; scape with black hairs, pedicel with circlet of black hairs; first flagellomere elongated, 1.92 times longer than wide, apically slightly sharp; arista black, indistinctly pubescent, basal segment 0.18 times as long as apical segment. Proboscis black with brown hairs; palpus black with brown hairs.

Thorax metallic green with pale gray pruinescence. Hairs and bristles on thorax black. Five strong dc, six acr, two strong npl, one strong sa, two strong pa; scutellum with two pairs of sc. Propleuron with one pale bristle. Legs mostly black. All coxae black; all femora black except apical 1/4 of fore femur yellow; all tibiae yellow except apical 1/3 of hind tibia black; fore and mid tarsi from tip of tarsomere I onward black, hind tarsus entirely black. Hairs and bristles on legs black. Fore coxa with eight bristles, mid coxa with six bristles, hind coxa with four outer bristles. All femora with two rows of long ventral bristles, twice as long as width of femur. Fore tibia with one ad, four pd, and three apical bristles; mid tibia with one ad, three pd, three av, and three apical bristles; hind tibia with five ad, six pd, av absent and four apical bristles. Fore tarsomere II slightly thickened with short thick bristles. Relative lengths of tibia and 5 tarsomeres LI 2.5 : 1.7 : 0.4 : 0.4 : 0.2 : 0.3; LII 3.0 : 1.4 : 0.7 : 0.5 : 0.3 : 0.2; LIII 2.8 : 1.0 : 0.5 : 0.4 : 0.3 : 0.2. Wing hyaline, veins black; costal callus indistinct; M bent medially, M and R_4+5_ parallel apically; CuAx ratio 0.48. Squama yellow with black hairs. Halter yellow.

Abdomen metallic green with pale gray pruinescence. Abdominal tergite II with large yellow lateral spot. Hairs and bristles on abdomen black. Male genitalia (Fig. 13): Surstylus deeply incised apically; dorsal lobe brown, nearly strip-like, with one apical bristle; ventral lobe yellow, finger-like, basally wide, with two bristles. Cercus long, medially wide, with distinct bristles. Hypandrium yellow, long, medially bent, apically rounded.

Female (Fig. 6). Body length 5.4–5.6 mm. Wing length 5.4–5.6 mm. Fore coxa yellow except basally black. Hind femur black apically; hind tibia yellow except apical 1/5 black. Fore tarsomere II normal without short thick bristles. Abdomen without yellow lateral spot.

#### Types.

Holotype male, CHINA, Sichuan, Pingwu, Wanglang National Nature Reserve, Baixionggou; 2857 m; collected by sweeping nets in grassland; 2016.VIII.06, Yuqiang Xi. Paratypes: 17 males, 448 females, same data as holotype.

#### Distribution.

Oriental realm: China (Sichuan).

#### Remarks.

The new species is somewhat similar to *A. beijingensis* Wang & Yang, but can be separated from the latter by the following features: all femora black, only fore femur yellow at apical 1/4; hind tibia apically black; fore tarsomere II slightly thickened with short thick bristles; dorsal lobe of surstylus apically unobviously inflated, nearly strip-like, with one apical bristle. In *A. beijingensis*, the ventral surface of the fore and mid femora are black at base, the hind femur is black at apical 1/5; the dorsal lobe of the surstylus is wide, apically distinctly inflated with three bristles (Wang et al. 2004: 386, fig. 2; Yang et al. 2011: 1106, fig. 714).

#### Etymology.

The specific name refers to the type locality, Sichuan province.

## Supplementary Material

XML Treatment for Argyra

XML Treatment for Argyra longicornis

XML Treatment for Argyra pingwuensis

XML Treatment for Argyra sichuanensis
